# Optical and electronic properties of MgPc-Ch-diisoQ blend organic thin film as an active layer for photovoltaic cells

**DOI:** 10.1371/journal.pone.0299079

**Published:** 2024-04-17

**Authors:** Marwah Ahmed Alsharif, A. A. A. Darwish, Saleem I. Qashou, Omaymah Alaysuy, E. F. M. El-Zaidia, S. A. Al-Ghamdi, M. Sadiq, Rania Saleh Alqurashi, Mohammad H. Al-Abandi, Taymour A. Hamdalla

**Affiliations:** 1 Faculty of Science, Department of Physics, University of Tabuk, Tabuk, Saudi Arabia; 2 Faculty of Science, Department of Physics, Zarqa University, Zarqa, Jordan; 3 Faculty of Science, Department of Chemistry, University of Tabuk, Tabuk, Saudi Arabia; 4 Faculty of Education, Department of Physics, Ain Shams University, Roxy, Cairo, Egypt; 5 Faculty of Science, Physics Department, Al-Baha University, Alaqiq, Saudi Arabia; 6 Faculty of Science, Physics Department, Alexandria University, Alexandria, Egypt; Mohanlal Sukhadia University, INDIA

## Abstract

Organic photovoltaic cells are a promising technology for generating renewable energy from sunlight. These cells are made from organic materials, such as polymers or small molecules, and can be lightweight, flexible, and low-cost. Here, we have created a novel mixture of magnesium phthalocyanine (MgPc) and chlorophenyl ethyl diisoquinoline (Ch-diisoQ). A coating unit has been utilized in preparing MgPc, Ch-diisoQ, and MgPc-Ch-diisoQ films onto to FTO substrate. The MgPc-Ch-diisoQ film has a spherical and homogeneous surface morphology with a grain size of 15.9 nm. The optical absorption of the MgPc-Ch-diisoQ film was measured, and three distinct bands were observed at 800–600 nm, 600–400 nm, and 400–250 nm, with a band gap energy of 1.58 eV. The current density-voltage and capacitance-voltage measurements were performed to analyze the photoelectric properties of the three tested cells. The forward current density obtained from our investigated blend cell is more significant than that for each material by about 22%. The photovoltaic parameters (*V*_*oc*_, *I*_*sc*_, and *FF*) of the MgPc-Ch-diisoQ cell were found to be 0.45 V, 2.12 μA, and 0.4, respectively. We believe that our investigated MgPc-Ch-diisoQ film will be a promising active layer in organic solar cells.

## 1. Introduction

Organic solar cells (OSCs) are regarded as the future source of energy because of their eco-friendliness, flexibility, ease of large-area fabrication, and low cost [1,2]. To achieve this goal, many new organic compounds with quite different properties have been examined to search for candidate materials to compete with the uses of silicon in the designing of photoelectrical devices. OSCs have made significant strides recently in terms of power conversion efficiency (PCE) and the creation of novel organic semiconducting materials and devices [3]. One of the suggested methods to improve photon harvesting of active layers was to use blends by adding an extra donor or acceptor to conventional binary blends [4].

Phthalocyanine and quinoline compounds are essential organic semiconductor materials that are characterized by their high thermal and chemical stability [5,6]. Phthalocyanine compound is a green-blue dye; the color comes from the high absorption in the UV-vis spectrum. Due to their high extinction coefficient in the red/near-infrared (NIR) region, phthalocyanines have attracted a lot of interest to be applied in OSCs in the past thirty years [7]. On the other line, the Quinoline compound is an aromatic heterocyclic compound that has a double-ring structure; its chemical formula is C_9_H_7_N. It is composed of a pyridine ring and benzene with two neighbor carbon atoms. Further, Quinoline is considered a distinct material to be fabricated with peripheral carbonyl groups since it can donate a lone pair of electrons [8]. The quinoline-phthalocyanine blend compound is involved with the desired structural and optical properties that play an important role in the enhancement of optoelectronic performance.

Metal phthalocyanines (MPcs) have been considered promising candidates due to their high absorption and thermal stability [9]. Moreover, MPcs have been widely acknowledged for the metal ion properties that are coordinated with other atoms or groups in the compound, which are important in tuning the properties of MPcs [9]. Therefore, Manganese Phthalocyanine (MgPc) is considered a significant complex material to be utilized in different photovoltaic applications [10,11]. MgPc compound has a high conductivity of charges due to the presence of Mg within phthalocyanine. MgPc’s electronic structure has been utilized in various photoelectric devices because of its strong light absorption in the visible region [12]. On the other way, Quinoline derivatives have been utilized for designing organic light-emitting diodes (OLEDs) in several research papers [13,14]. Among quinoline derivatives, 2,9-Bis[2-(4-chlorophenyl)ethyl]anthrax[2,1,9-def:6,5,10-d0e0f0]diisoquinoline-1,3,8,10 (2H, 9H) tetrone (Ch-diisoQ) is considered as one of the promising materials that can be used for the manufacturing of solar cells units due to the wide range of light absorption [15,16]. Xu et al conducted a study on enhancing polymer solar cells by adding a smaller amount of donor material into the acceptor layer [17]. This improvement is achieved by increasing the efficiency of utilizing excitons in the acceptor layer near the electrode. Zhang et al emphasize the importance of functional layer materials in recent advancements in efficient polymer solar cells. These materials include polymer donors, novel polymer acceptors made by polymerizing small molecule acceptors, interfacial materials, and rational design principles for these functional materials [18].

Recently, there have been many studies on the synthesis of a phthalocyanine-quinoline matrix to examine the ability to use such a new fabricated matrix in different electrical and optical field applications. Cobalt (II) tetra methyl-quinoline-phthalocyanine, for example, has been fabricated and characterized to be used in sensing nitrite [19]. It was discovered that the aromatic quinoline ring with the methyl substitution increased the conjugation effect, which enhances the effectiveness of nitrite detection [19]. Ahmetali *et al*. synthesized zinc phthalocyanine (ZnPc) that is replaced with 8-hydroxyquinoline and contains two active chelating donor atoms [20]. They conclude that the new ZnPc may hold promise as photosensitizers for photodynamic therapy treatment due to their appropriate photochemical and photophysical parameters [20].

**Scheme 1:**
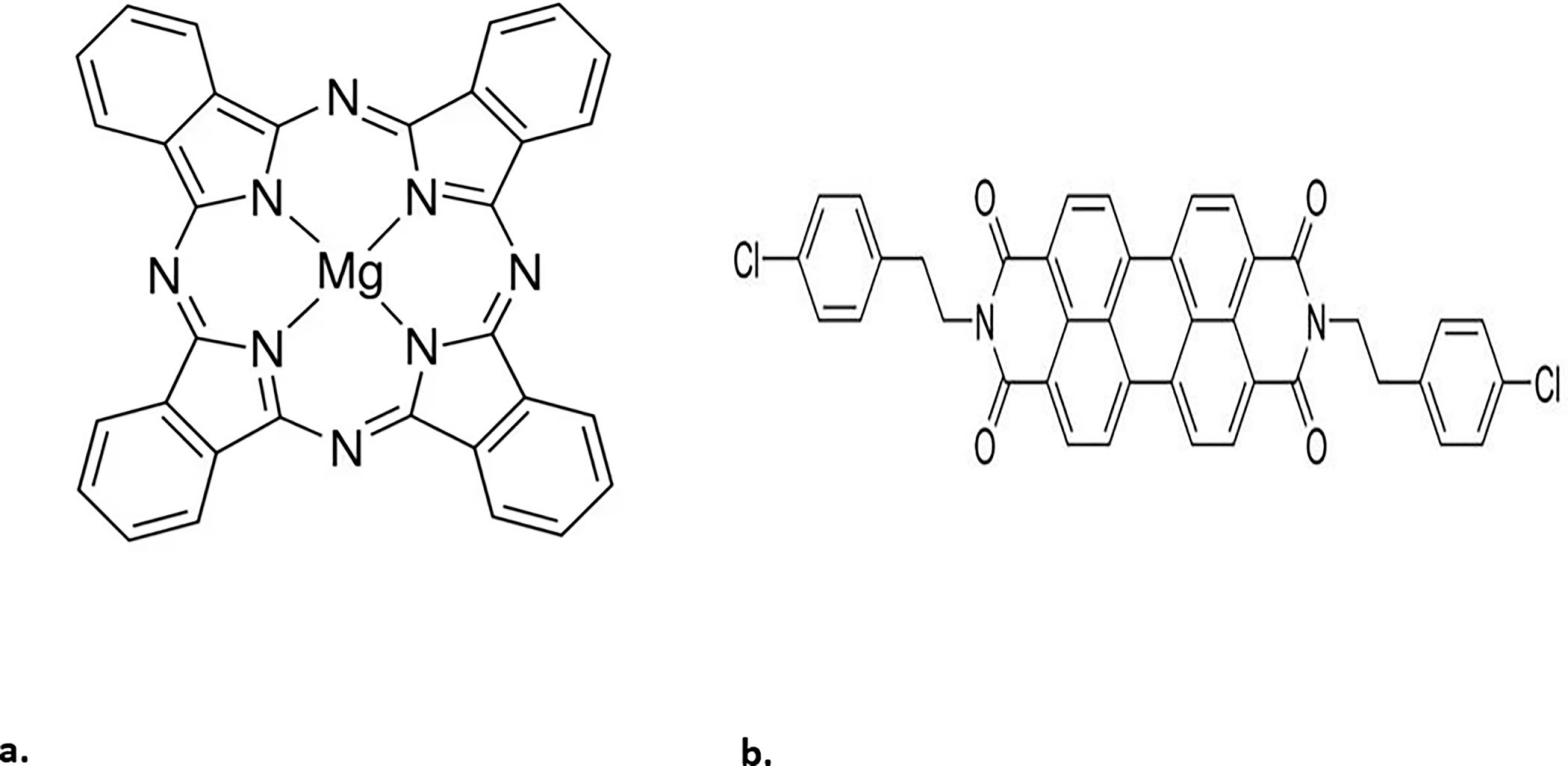
The chemical structure of **(a)** MgPc and **(b)** Ch-diisoQ compounds.

This study presents for the first time the combination of MgPc and Ch-diisoQ as a blend. **Scheme 1 shows t**he chemical structure of **(a)** MgPc and **(b)** Ch-diisoQ compounds. The morphology, optical, and photoelectric properties of the deposited MgPc, Ch-diisoQ, and MgPc-Ch-diisoQ on the conductive FTO substrates have been investigated. The capacitance-voltage (*C*-*V*) and current density-voltage (*I*-*V*) characteristics under operating conditions have been investigated. We believe that MgPc-Ch-diisoQ thin films will have great resonance in improving the performance of the solar cell units.

## 2. Experimental details

MgPc powder **(**Dye content 90%, Molecular Weight:536.83), and Ch-diisoQ powder (purity: 94%, Molecular Weight: 309.198) have been purchased from Sigma-Aldrich Company. The identical weight (500 mg) of MgPc and Ch-diisoQ powders was weighed individually using a sensitive electronic balance. Then they were mixed with mortar and pestle. MgPc-Ch-diisoQ has been grind by using a ball mill for 30 min to make the mixture powder. The motivation behind using the ball milling technique in preparing a blend is mainly to achieve a uniform and well-dispersed mixture of materials. Ball milling involves placing materials into a grinding vessel along with small metal balls, which are then agitated to create a collision between the balls and the materials. This collision effectively grinds and mixes the materials, leading to better dispersion and homogeneity. And to ensure efficient charge transport and effective light absorption. After that, the mixture powder is put in a microwave oven for 12 h to be linked.

**Scheme 2:**
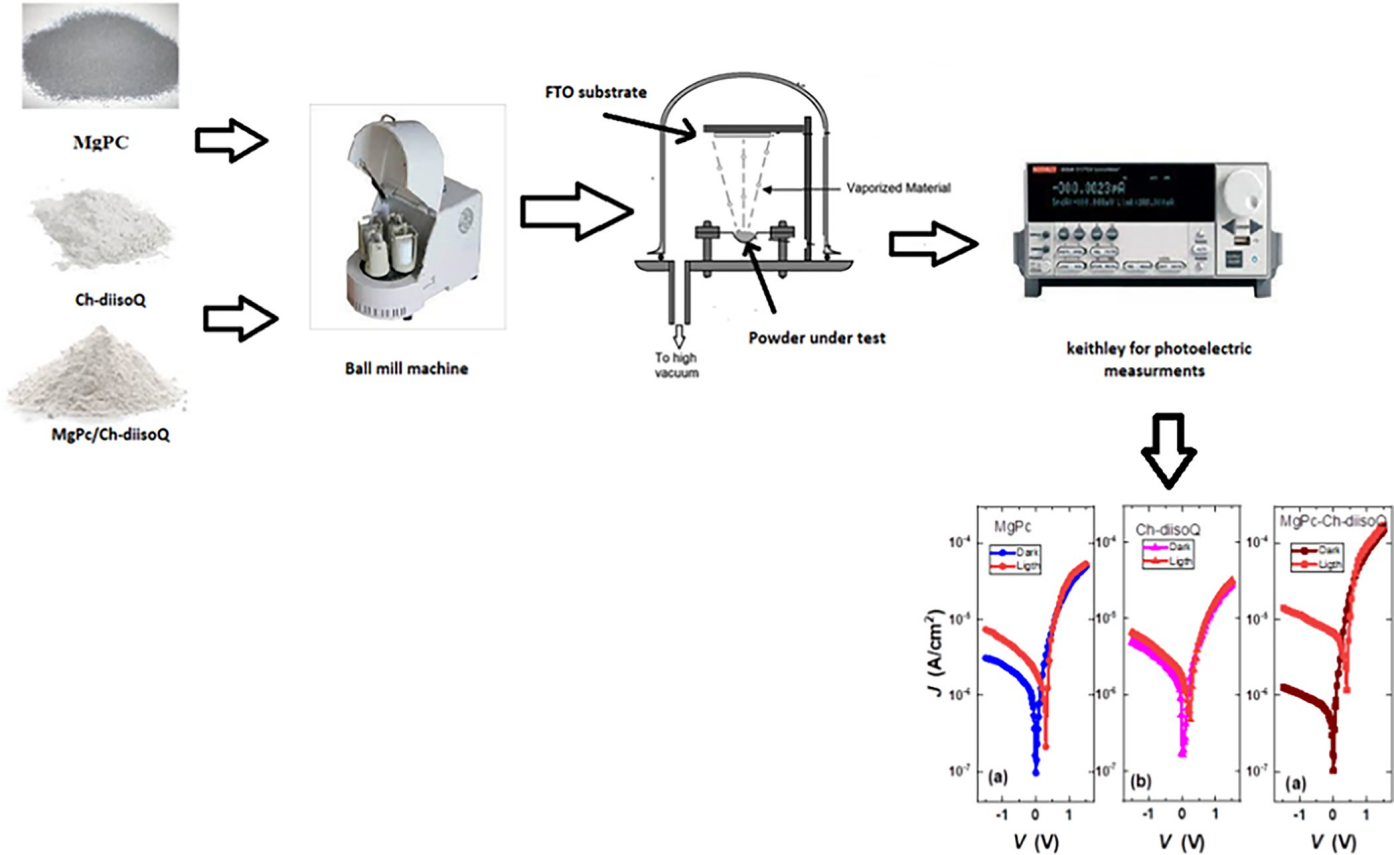
The experimental setup for the preparation of investigated films.

Separately, MgPc, Ch-diisoQ, and MgPc-Ch-diisoQ thin films were created by utilizing an Auto HHV 306 high vacuum coating unit. At a pressure of 3×10^−4^ Pa, MgPc, Ch-diisoQ, and MgPc-Ch-diisoQ thin films were deposited on exceptionally cleaned FTO/glass substrates. The thickness (70 nm) of our three investigated thin films is monitored by the attached monitor with a deposition rate of 7 Å/s. Subsequently, the top electrode of Al was deposited by usual thermal evaporation to format a cell with an active area of 0.78 cm^2^. Scheme 2 shows the experimental setup of our work.

An ATI Mattson FTIR spectrophotometer was utilized to acquire Fourier-transform infrared (FTIR) spectra. The surface morphology of the three prepared films has been examined using a scanning electron microscope (SEM). To prepare our investigated films to get SEM images, the examined film was fitted with a coated carbon tap. Then, it was given a thin layer of gold coating using an ion-sputtering coating system while being vacuumed. Images from the JEOL-JFC-1100E SEM were captured at 25 kV. The X-ray diffraction (XRD) pattern was utilized to examine the deposited thin film of MgPc, Ch-diisoQ, and MgPc-Ch-diisoQ utilizing a Philips X-ray diffractometer (model X’ Pert) with the following operating conditions. The initial and final diffraction angles were 2θ = 4° and 2θ = 80°, respectively; meanwhile, CuKα radiation turned on at 25 mA and 40 kV. The absorption spectra of all prepared films were measured by using a double beam spectrophotometer Jenway model 6800 UV/Vis at the wavelength range 190–1100 nm, while the resolution was around ± 0.1 nm.

The C-V characteristics were examined using a Hioki 3536 Hitester automatic RLC bridge. The current density flowing through the cells was measured using a Keithley 2635B electrometer and source meters. The three cells were subjected to continuous white light from a halogen lamp (FO7 Philips 6423), and the J-V measurements were taken with an illumination intensity of 80 mW/cm2, regulated by a solar power meter (TM206). It is important to mention that all measurements were conducted in ambient air at room temperature.

## 3. Results and discussions

### 3.1. Structural studies

**Fig 1:**
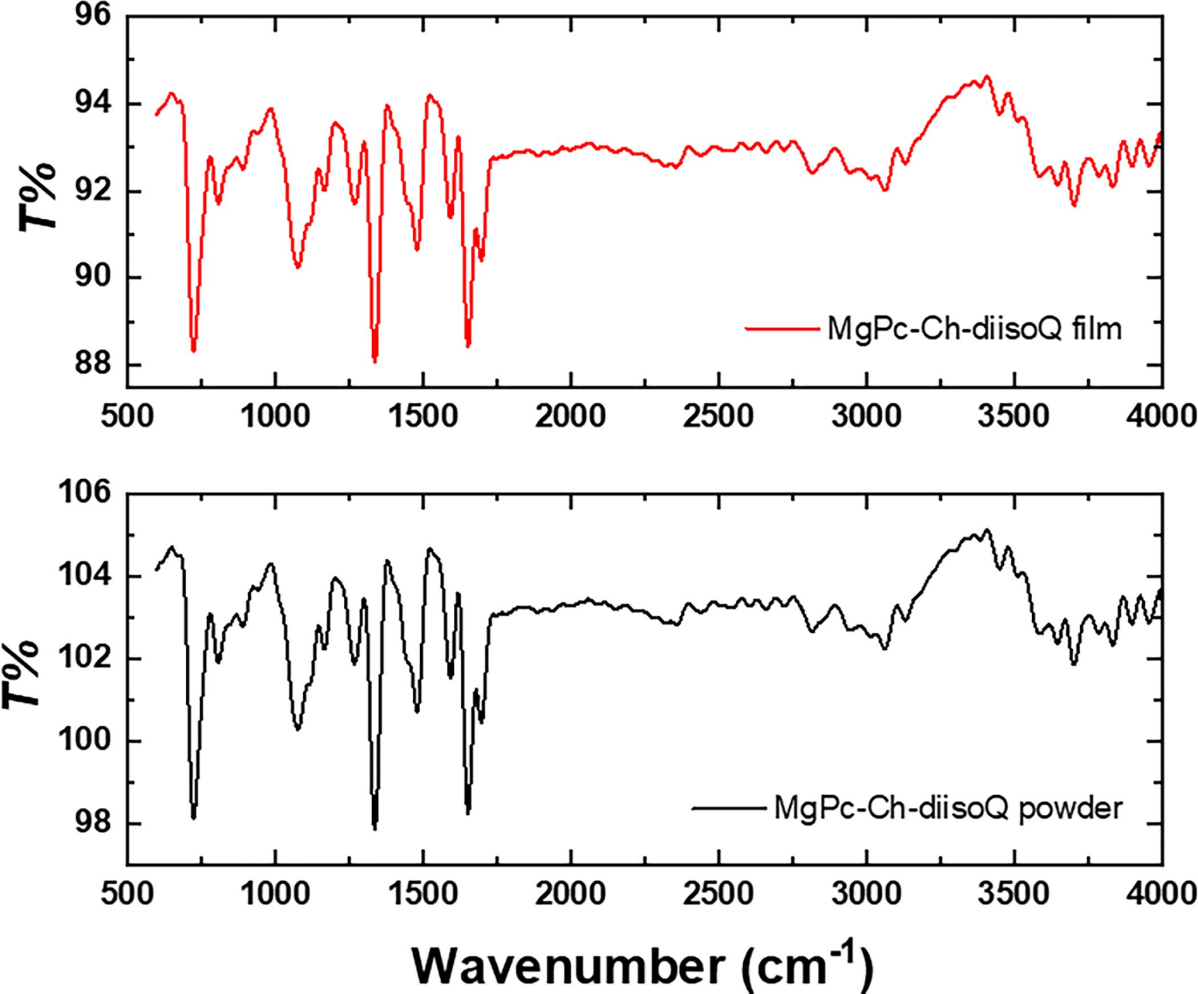
FTIR spectra of MgPc-Ch-diisoQ powder and film.

The organic structure can be determined by analyzing the interatomic bonds’ absorption using FTIR spectroscopy. Fig 1 displays the FTIR spectra of MgPc-Ch-diisoQ powder and films in the range of 500 cm^-1^ to 4000 cm^-1^. The FTIR spectrum of the as-deposited film remained unchanged after evaporation, indicating that this method is useful for obtaining undissociated MgPc-Ch-diisoQ films.

**Fig 2:**
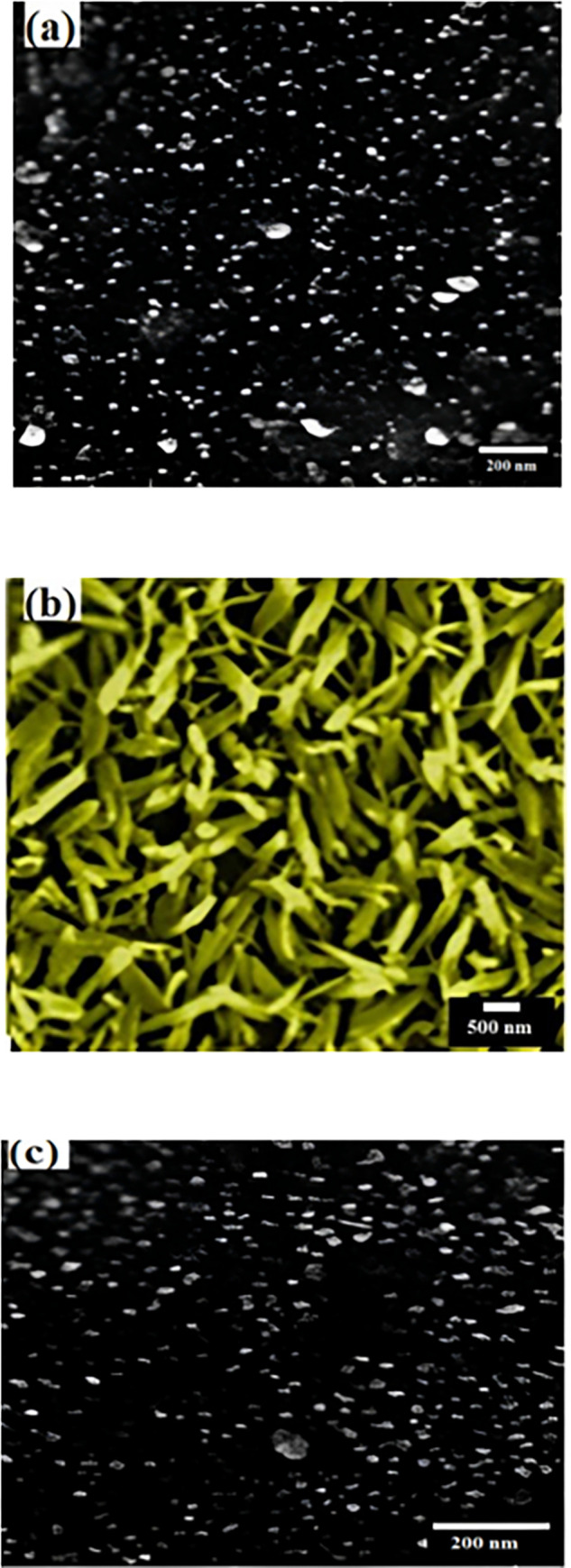
The SEM images of **(a)**MgPc, **(b)**Ch-diisoQ, and **(c)**MgPc-Ch-diisoQ thin films.

SEM is a familiar technique that is usually used by researchers to study the material’s surface, especially nanoparticles. Fig 2 shows the SEM images of MgPc, Ch-diisoQ, and MgPc-Ch-diisoQ thin films. It can be seen from the SEM images that the distribution of pure MgPc film is characterized by its unique spherical and homogeneous shape. The surface morphology of pure Ch-diisoQ film is like tree leaves (rod shapes). MgPc-Ch-diisoQ films, on the other hand, have remarkably similar morphology to pristine MgPc films. All the grain size measurements have been done using Image J software. The average grain size of MgPc/FTO and MgPc-Ch-diisoQ/FTO was 15.6 nm and 15.9 nm, respectively. The average width is 131.8 nm, and the length of nanorods is about 810 nm for Ch-diisoQ/FTO. The similarity in surface morphology between thin films of the organic blend MgPc-Ch-diisoQ and pristine MgPc films could be due to several factors. One possible reason is that the Ch-diisoQ component, despite being blended with MgPc, may not significantly alter the surface characteristics. Furthermore, during the film formation, the molecules of MgPc and Ch-diisoQ could arrange themselves in a way that maintains a similar morphology to the pristine MgPc films. This could occur due to molecular self-assembly or similar intermolecular interactions between the two components.

**Fig 3:**
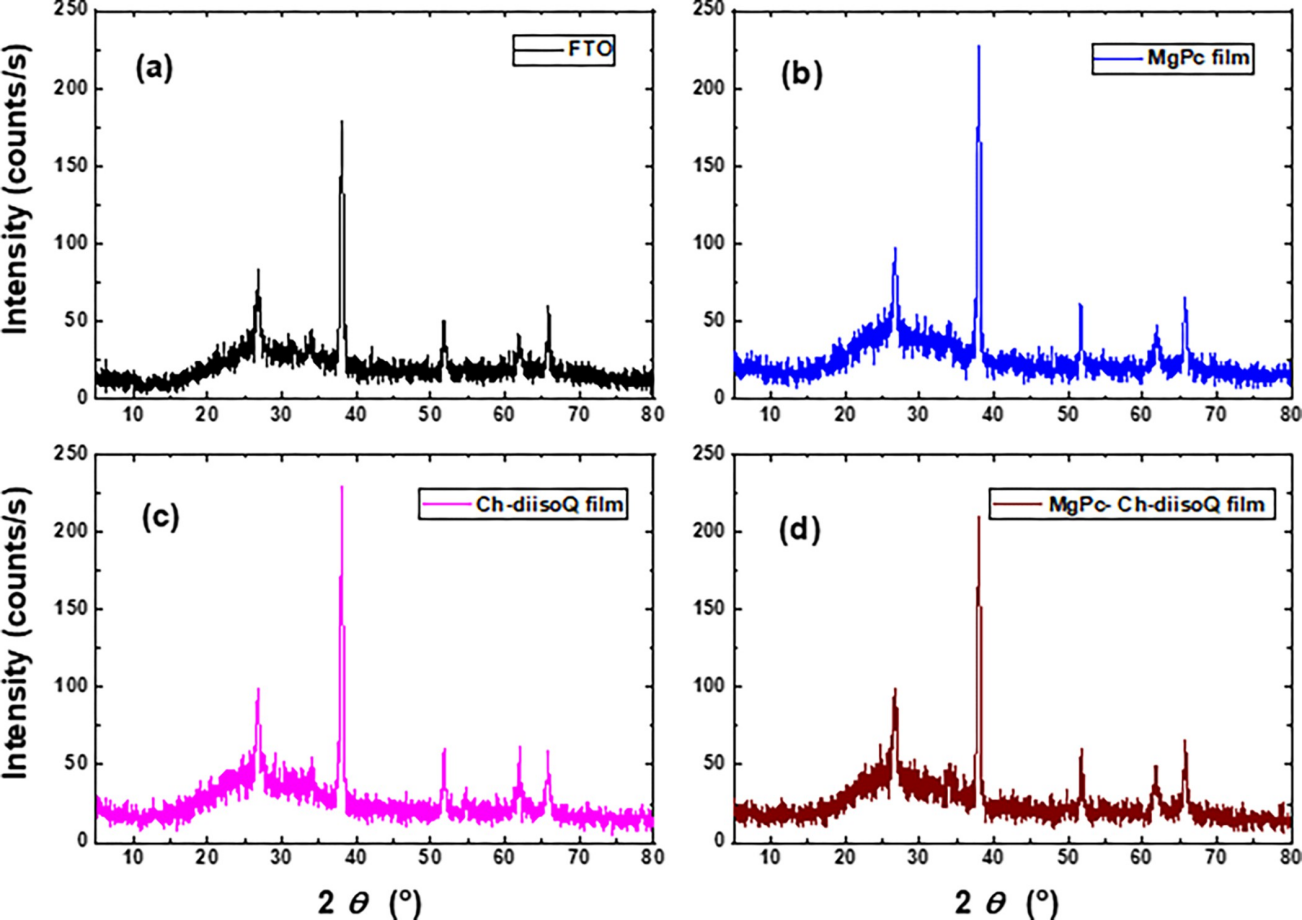
XRD pattern of **(a)** FTO, **(b)** MgPc, **(c)** Ch-diisoQ, and **(d)**MgPc-Ch-diisoQ films.

The crystal structure of our investigated materials was examined using XRD measurement. Fig 3 depicts the pattern of XRD for FTO, MgPc, Ch-diisoQ, and MgPc-Ch-diisoQ thin films. All the XRD patterns have the same five diffraction peaks. These peaks correspond to the crystalline planes of the FTO substrate, as reported in previous publications [21,22]. According to the XRD Fig, at 2θ° equal to 28.6°, 38.7°, 51.9°, 61.5° and 66.8° are corresponding to (110), (101), (200), (310) and (301). Thus, we conclude that the three deposited materials on the FTO substrate are amorphous. From our review of previous studies of our investigated materials, we found that they are an amorphous structure [15,23,24]. As previously reported [21,22,25–27], most of the organic materials are amorphous at room temperature when they are deposited on an FTO glass substrate. The high stack of three films on the surface of FTO is supported by the XRD results, which enhance the morphology of the film surface on the FTO layer.

### 3.2. Optical properties

**Fig 4:**
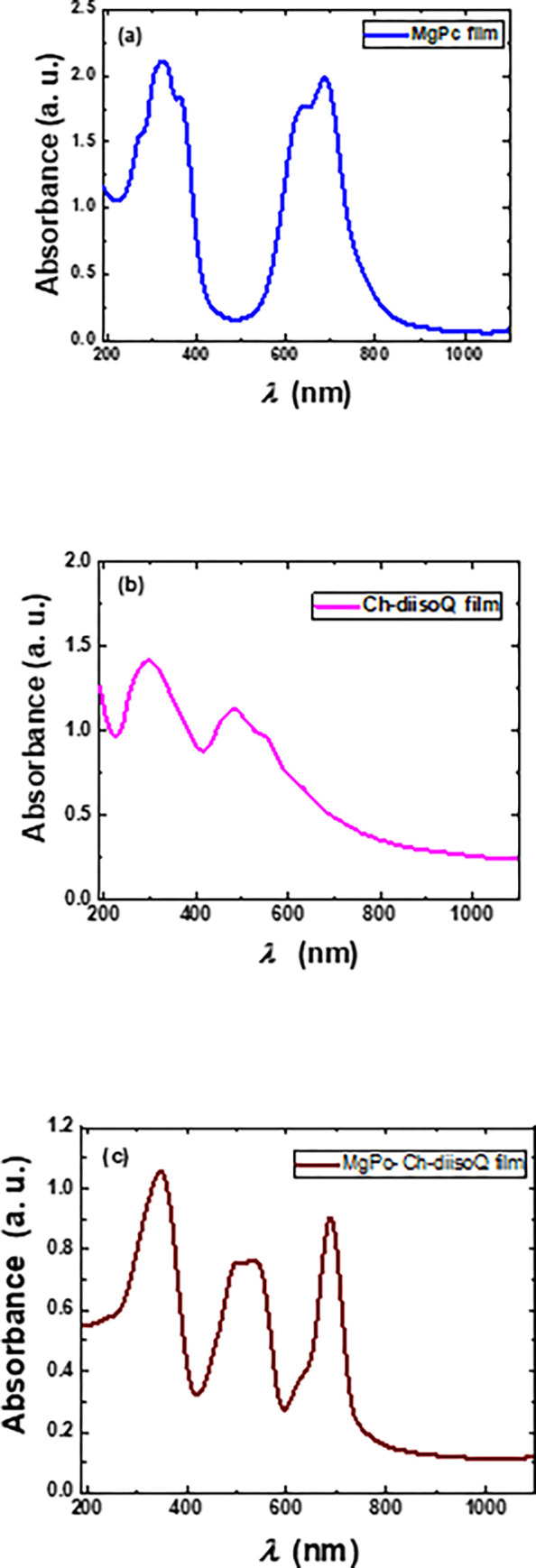
Absorbance spectra of **(a)** MgPc, **(b)** Ch-diisoQ, and **(c)** MgPc-Ch-diisoQ films.

The recent optoelectronic techniques and advanced materials selection both depend on optical absorbance (A). Fig 4 shows the absorbance spectra of MgPc, Ch-diisoQ, and MgPc-Ch-diisoQ thin films. Fig 4A depicts the absorption spectrum of MgPc film. MPcs’ UV-vis spectrum is derived from aromatic 18-electron molecular orbitals and overlapping orbitals on the central metal atom [28]. The Q-band, a visible absorption band, has been attributed to π–π* transitions on the phthalocyanine macrocycle and appears between 600 and 800 nm. The electronic transition from π–π* causes an intense band in the UV spectral region (400–200 nm) known as Soret (B-band). On the other hand, Fig 4B displays the absorption of Ch-diisoQ film, which also has two absorption regions: The first domain at 600–400 while the second region at 400–200, which is because of the electronic transition from π–π*. As noted in Fig 4C, the MgPc-Ch-diisoQ film has three significant absorption bands at 800–600 nm, 600–400 nm, and 400–250 nm. Therefore, the band at 600–400 nm is significant in the fabricated blend. This absorption band has appeared because of the blending of MgPc and Ch-diisoQ. It is created due to the cross-linking between MgPc and Ch-diisoQ after being ground and mixed using the mechanical ball mill. Additionally, the position of this band falls within the visible light range (400–700 nm), making it easily detectable and relevant for practical applications. Being in the visible region means that it interacts with the light we can see, which could potentially result in coloration or other visually detectable properties. Furthermore, the blend composite could lead to the formation of a charge transfer complex between the two species. This could result in the appearance of a new absorption band at a different wavelength than the individual species would absorb.

Tauc’s method [29] can be used to calculate the band gap energy (*E*_*g*_) in the vicinity of the absorption edge. The absorption coefficient, *α*, is calculated from the relation [30]:

$$\alpha =\frac{2.303\times A}{d}$$ (1)

*d* is the film thickness in cm.

**Fig 5:**
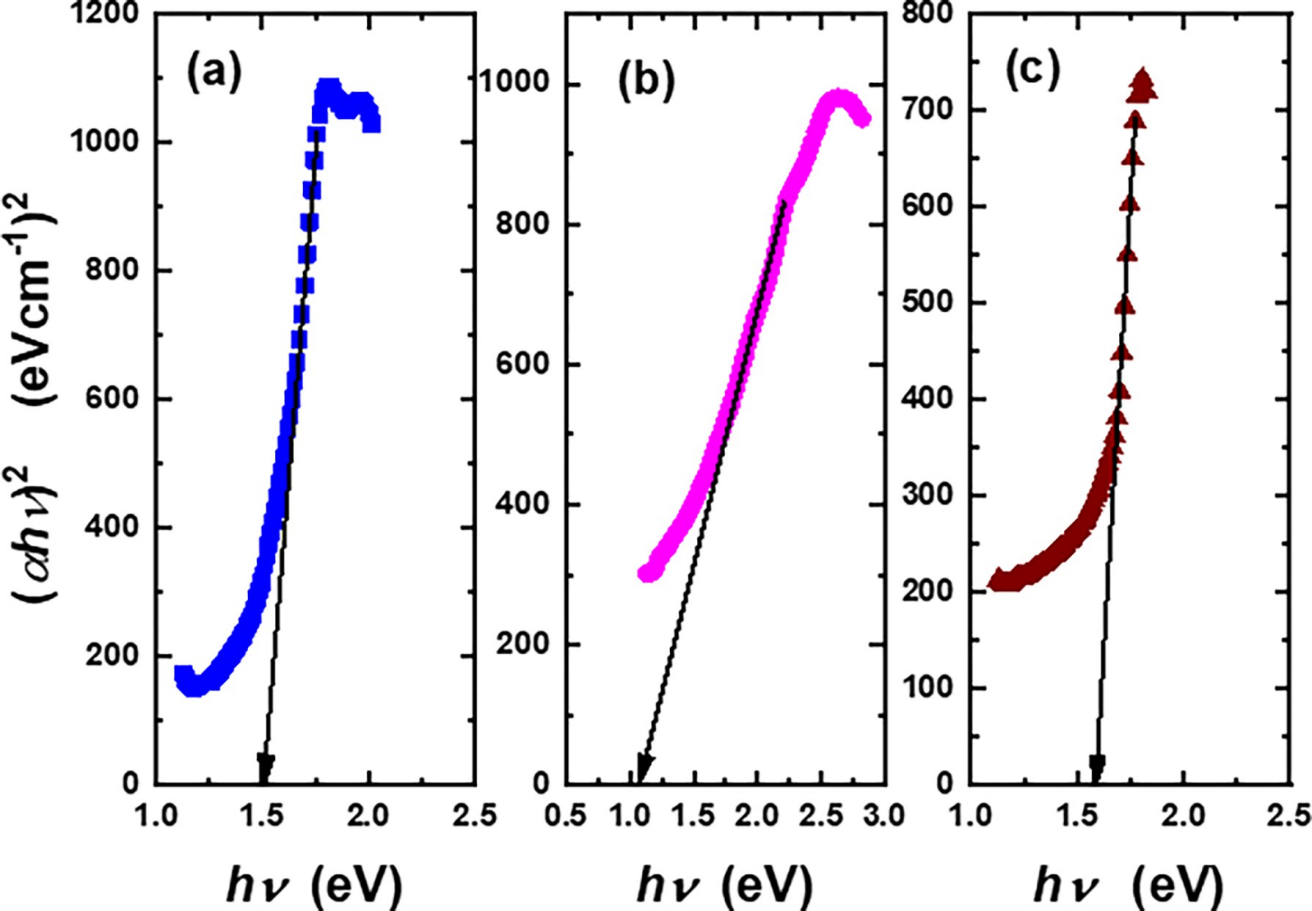
Relation between (*αhν*)^2^ and *hν* for **(a)** MgPc, **(b)** Ch-diisoQ, and **(c)** MgPc-Ch-diisoQ films.

Additionally, the Tauc plot method is relatively simple and easy to use, making it a popular technique for measuring the bandgap energy of materials. Therefore, the Tauc plot method is a preferred method for measuring the bandgap energy of materials in various research and industrial applications. The main advantage of using the Tauc plot method for measuring the bandgap is that it provides a more accurate determination of the bandgap energy compared to other methods. The Tauc plot method applies to a wide range of materials, including both direct and indirect bandgap materials. The energy bandgap could be calculated using [29]:

$$\left({\alpha h\nu )}^{K}=Z(\alpha h\nu -E_{g}\right)$$ (2)

where *Z* is the band tailing parameter, *hν* is photon energy, and *K* is the quality factor that governs the electronic transition, which is usually determined from *K* values. If *K* equals ½, then the indirect-allowed is dominant, while if *K* equals 2, then the direct-allowed is predominant [29]. Fig 5 shows the variation of (*αhν*)^1/2^ with *hν* for MgPc, Ch-diisoQ, and MgPc-Ch-diisoQ films. According to Eq 2, *E*_*g*_ is the intersection of the linear part with the *hν*-axis. As noted in Fig 5, The values of *E*_*g*_ of MgPc, Ch-diisoQ, and MgPc-Ch-diisoQ are 1.51, 1.08, and 1.58 eV, respectively. The value of *E*_*g*_ for MgPc-Ch-diisoQ has registered the highest value, which could be related to a decrease in disorder and the localized states within our investigated blend.

### 3.3. Photoelectric studies

**Fig 6:**
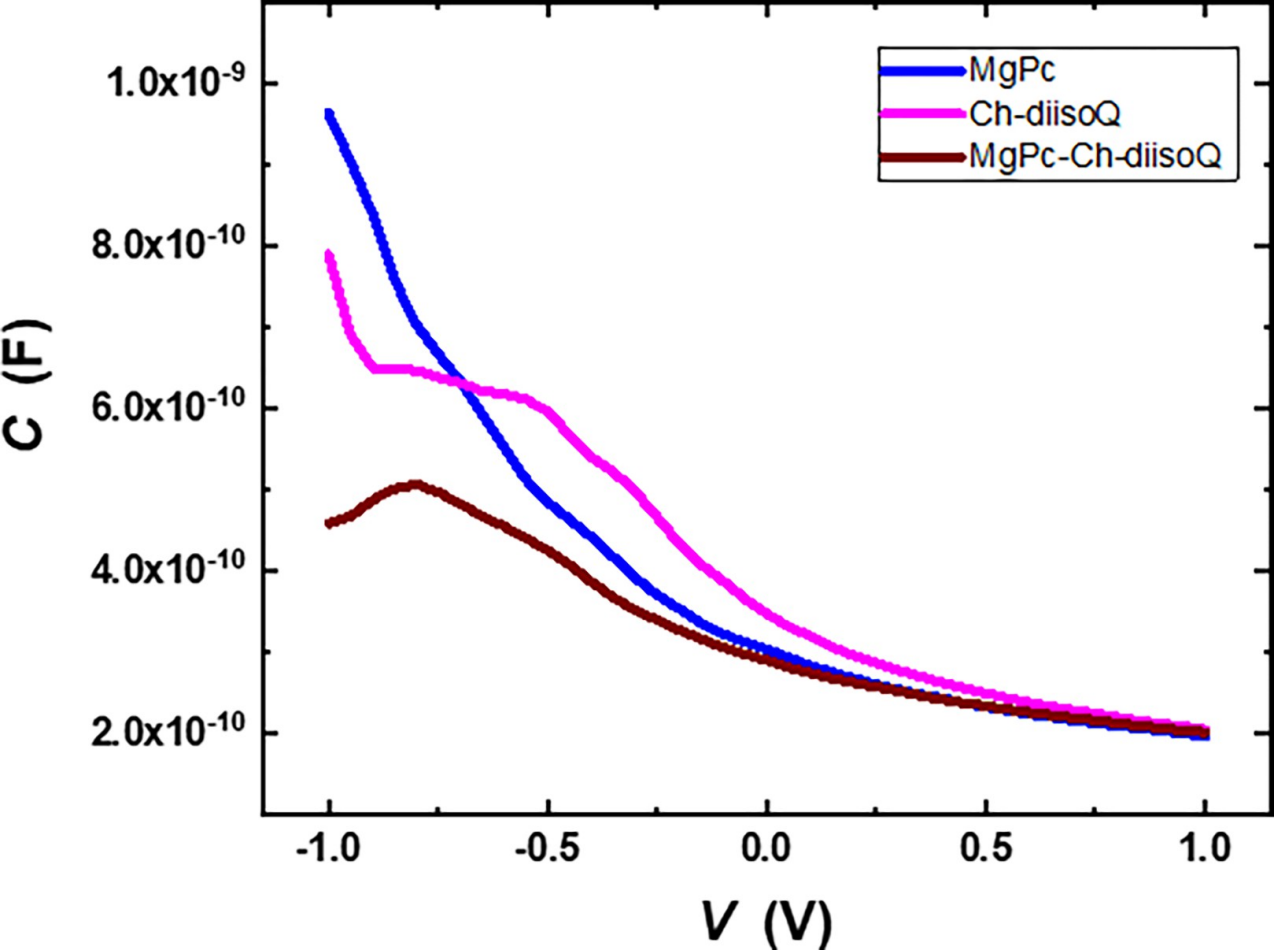
Variation of capacitance with voltage for FTO/MgPc/Al, FTO/Ch-diisoQ/Al, and FTO/MgPc-Ch-diisoQ/Al cells.

Capacitance is an important source of describing the behavior of the optoelectronic properties of materials. So, *C*-*V* measurements of the thin films under test have been done at a frequency of 1 MHz. This frequency is high enough to allow the dielectric relaxation process to be ignored [31]. Fig 6 shows the dark *C*-*V* measurements of the tested thin films, and we can notice that the capacitance of the MgPc-Ch-diisoQ/FTO shows an obvious reduction in the capacitance at the negative potential. This could be due to an increase in the energy band gap that acts to decrease the number of free charges. Also, the values of capacitance at zero voltage were calculated and listed in Table 1.

**Table 1:** Diode parameters of the three examined thin films extracted from dark *C*-*V* and *I*-*V* characteristics.

	*C*_*o*_ (10^−10^ F)	*V*_*bi*_ (V)	*RR*	*R*_*s*_ (kΩ)	*R*_*sh*_ (MΩ)	*n*	*J*_*o*_ (A/cm^2^)
MgPc/FTO/Al	3.04	0.69	10.73	40.6	0.65	2.13	9.25×10^−8^
Ch-diisoQ /FTO/Al	3.47	0.54	4.06	71.6	0.41	4.05	1.64×10^−7^
MgPc/Ch-diisoQ /FTO/Al	2.91	0.92	72.51	13.7	1.57	2.28	9.88×10^−8^

**Fig 7:**
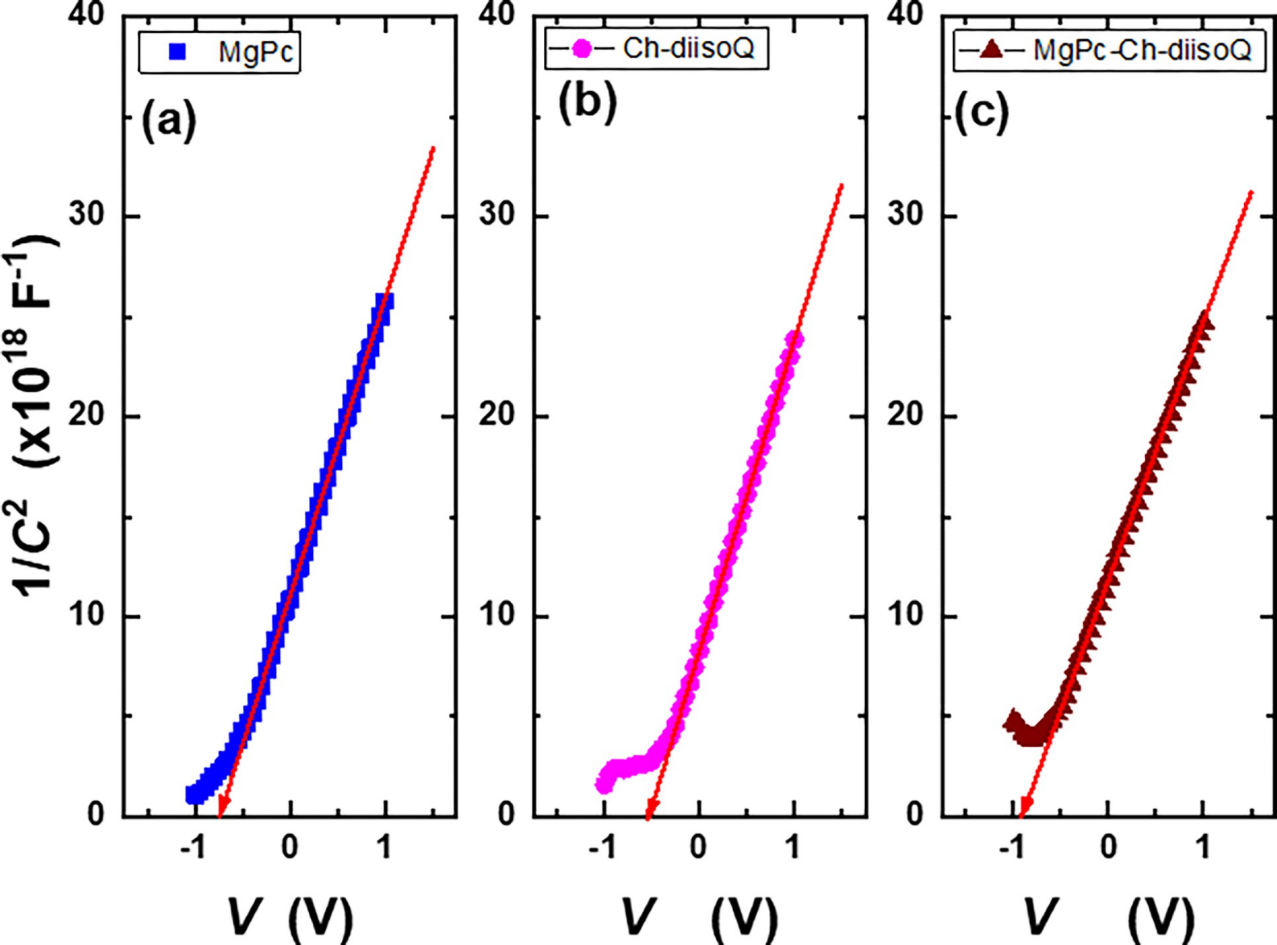
Variation of 1/*C*^2^ with *V* for **(a)** FTO/MgPc/Al, **(b)** FTO/Ch-diisoQ/Al, and **(c)** FTO/MgPc-Ch-diisoQ/Al cells.

The variation of 1/*C*^2^ with *V* for the prepared cells is represented by [5]:

$$\frac{1}{C^{2}}=-\frac{2}{q\;{\varepsilon_{s}\varepsilon }_{o}A^{2}N}(V_{b}-V)$$ (3)

where *V*: the applied voltage, *ε*_*s*_: is the dielectric constant, *q*: the electronic charge, *V*_*b*_: the built-in voltage, *N*: the carrier concentration, and *A*: the effective area. Fig 7 displays the relation between 1/*C*^2^ and *V* for three examined cells. It can be noticed from Eq 3 that the value of *V*_*b*_ could be calculated from the interception of the drawn straight line. It can be seen from Table 1 that the *V*_*b*_ values were 0.69 V, 0.54 V, and 0.92 V for MgPc, Ch-diisoQ, and MgPc-Ch-diisoQ, respectively. The high built-in voltage values of the blend are a good indication of the high open circuit voltage; this could nominate our blend as a promising active layer in OSCs.

**Fig 8:**
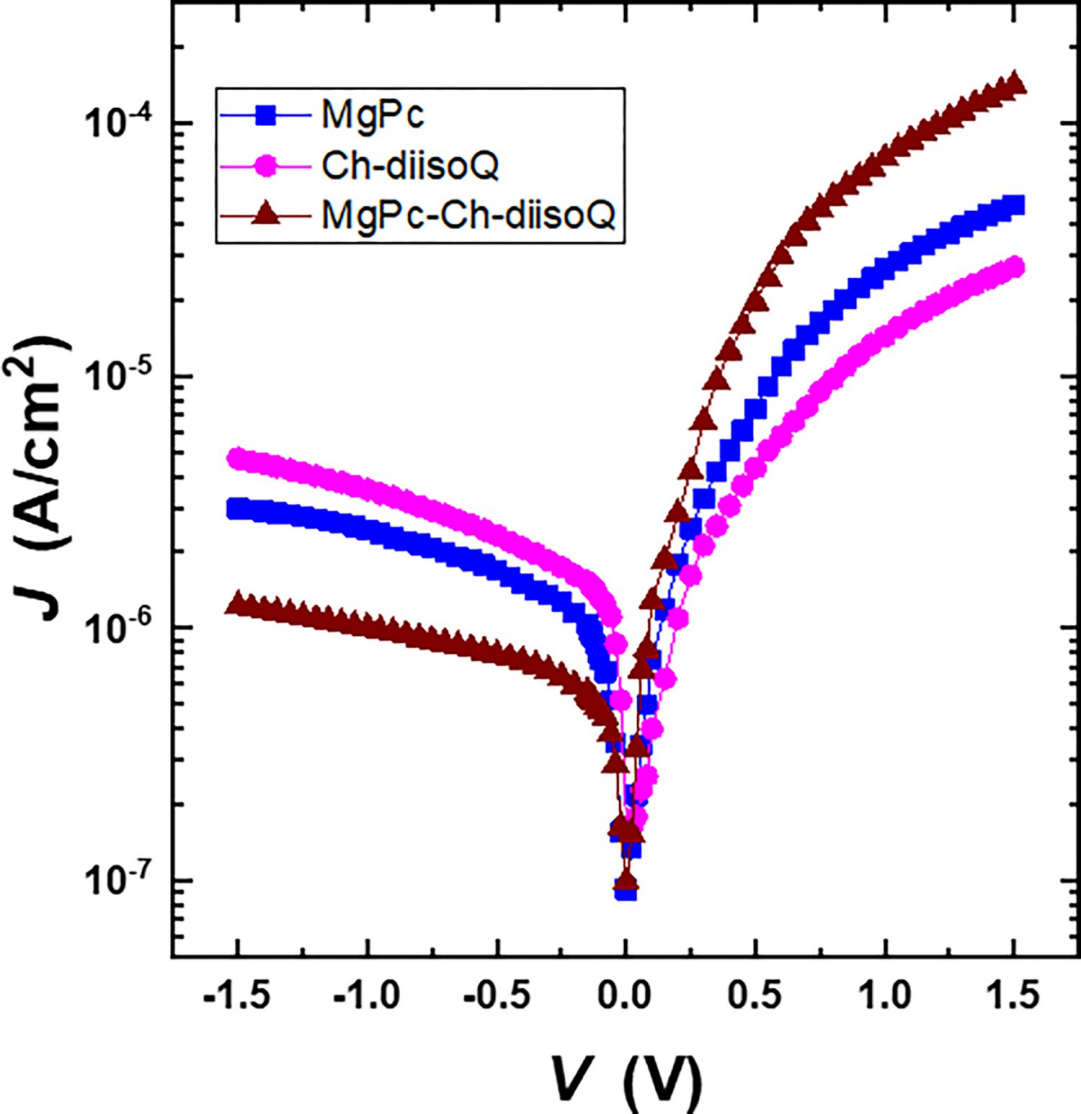
The dark *J*-*V* characteristics for FTO/MgPc/Al, FTO/Ch-diisoQ/Al, and FTO/MgPc-Ch-diisoQ/Al cells.

Fig 8 shows *J*-*V* characteristics under dark conditions for MgPc/FTO, Ch-diisoQ/FTO, and MgPc-Ch-diisoQ/FTO cells. We can conclude that the forward current density shows voltage dependence, and the maximum current density obtained from the blend cell is greater than the other cells by about 22%. The increase in the current density by 22% nominated the MgPc-Ch-diisoQ/FTO cell as an excellent rectification diode. The rectification ratio, *RR*, at +1 and– 1 V for the three cells was estimated and listed In Table 1. The current density is calculated using the following equation from the thermionic emission process [32]:

$$J=\left[J_{o}(\frac{q(V-JR_{s}}{nkT})-1\right]+\frac{V-JR_{s}}{R_{sh}}$$ (4)

where *J*_*o*_, *T*, and *n* are the reverse saturation current density, the absolute temperature, and the diode’s ideality factor, respectively. Also, *R*_*s*_ and *R*_*sh*_ are series and shunt resistances, respectively, which can be obtained from junction resistance (*R*_*j*_). Details about the diode’s performance can be found in Eq 4. The interfacial layer and/or the effect of *R*_*s*_ may both contribute to deviations in *J*-*V* features from linearity [33]. Furthermore, the low *R*_*sh*_ might make the leakage current density worse. The *R*_*s*_ and *R*_*sh*_ values of the diode are decisive for the conception of the forward *J*-*V* characteristics. The *R*_*j*_ can be calculated by using [32].


$$R_{j}=\frac{dV}{dI}$$ (5)

The diode resistances (*R*_*s*_ and *R*_*sh*_) for the three examined cells were determined and listed in Table 1. This table shows a decline in the blend cell’s *R*_*s*_ value, which could be expounded by a decline in the localized state’s stimulating interface charge mobility, as will become clear later from the analysis of space-charge-limited (SCLC). Furthermore, the forward current density axis’ straight-line intercept estimates the value of *J*_*o*_, and *n* represents the calculated diode quality factor [34]:

$$n=\frac{q}{kT}\left(\frac{dV}{d(lnJ)}\right)$$ (6)

The values of *J*_*o*_ and *n* of the three cells were estimated and recorded in Table 1. The actuality that the value of *n* is higher than one indicates the presence of a recombination current density driven by traps [35].

**Fig 9:**
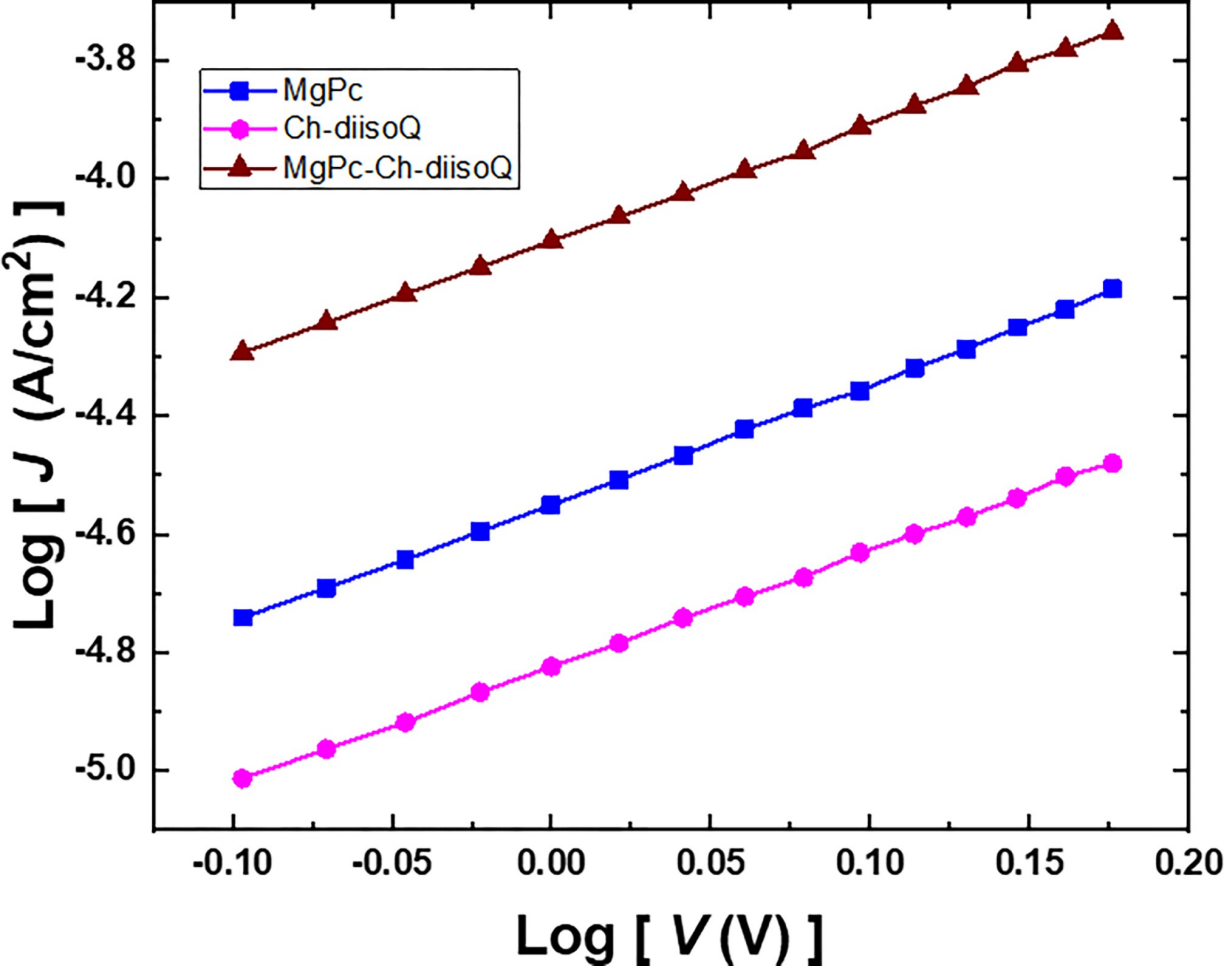
Double logarithmic scale of J-V characteristics for FTO/MgPc/Al, FTO/Ch-diisoQ/Al, and FTO/MgPc-Ch-diisoQ/Al cells.

In addition, by analyzing the J-V characteristics, we can determine the charge transport mechanisms in the three cells MgPc/FTO, Ch-diisoQ/FTO, and MgPc-Ch-diisoQ/FTO when the voltage exceeds 1 V. Fig 9 displays the *J*-*V* double log-log plot for the three cells, which displays straight lines with a slope of 2.02. This slope signifies a power dependency and suggests the presence of a single trap level mechanism with SCLC. The equation can be utilized to calculate the current in SCLC [36].


$$J=\left(\frac{9}{8}\right)\left(\frac{V^{2}}{d^{2}}\right)\left(\frac{N_{v}}{N_{t}}\right)\mu \;\varepsilon \varepsilon_{o}\;\exp\left(\frac{-E_{t}}{kT}\right)$$ (7)

This equation used to determine the current SCLC is defined by the variables *μ* (the mobility of charge carrier), *N*_*t*_ (the trap concentration at an energy level *E*_*t*_ below the conduction band edge), *ε*_*o*_ (the free space permittivity), and *ε* (the dielectric constant of the film). The SCLC model was further employed to investigate mobility of charge carrier [23], and our calculations of *μ* in the three cells MgPc/FTO, Ch-diisoQ/FTO, and MgPc-Ch-diisoQ/FTO were found to be 3.56×10^−7^, 2.71×10^−9^, and 5.34×10^−5^ cm^2^V^-1^s^-1^, respectively. The enhanced *μ* in MgPc-Ch-diisoQ/FTO cell should be beneficial to suppress charge recombination and improve charge collection, resulting in the relatively large photovoltage parameters for the MgPc-Ch-diisoQ/FTO cell.

**Fig 10:**
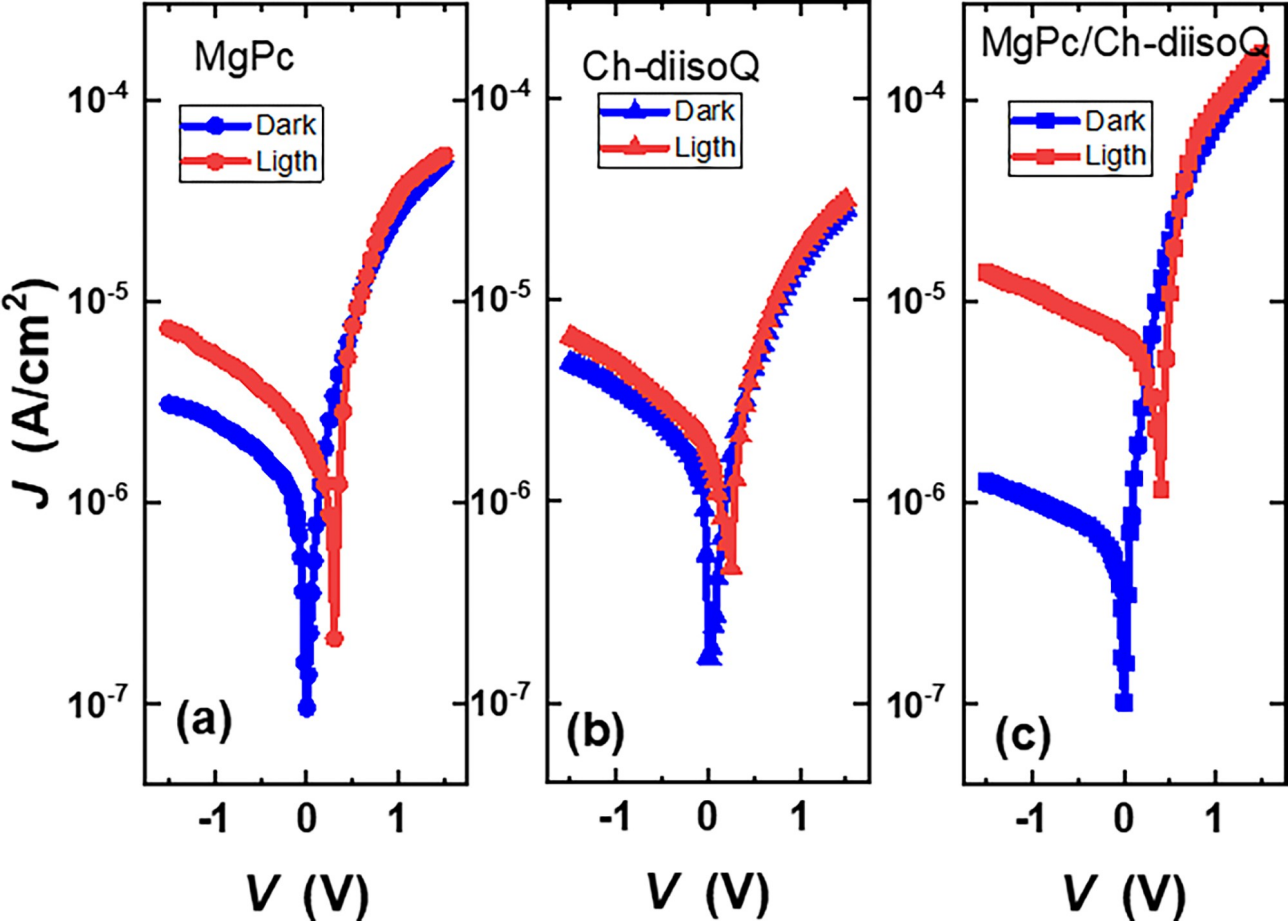
J-V characteristics (dark and light conditions) for (a) FTO/MgPc/Al, (b) FTO/Ch-diisoQ/Al, and (c) FTO/MgPc-Ch-diisoQ/Al cells.

The effect of illumination on the electronic properties of the three cells has been investigated. Fig 10 depicts the *J*-*V* characteristics of three cells MgPc/FTO, Ch-diisoQ/FTO, and MgPc-Ch-diisoQ/FTO in forward and reverse bias under illumination (80 mW cm^-2^) and at room temperature. For the three cells, the estimated current density at a given voltage is higher in the light than in the dark, as shown in Fig 10. The findings demonstrate that the formation of electron-hole pairs (excitons) and their subsequent dissociation into free charge carriers at the barrier cause carrier-contributing photocurrent to be produced by light absorption by the active layer [11]. In addition, the photocurrent value of the blend cell is high as a result of improved optical absorption.

**Fig 11:**
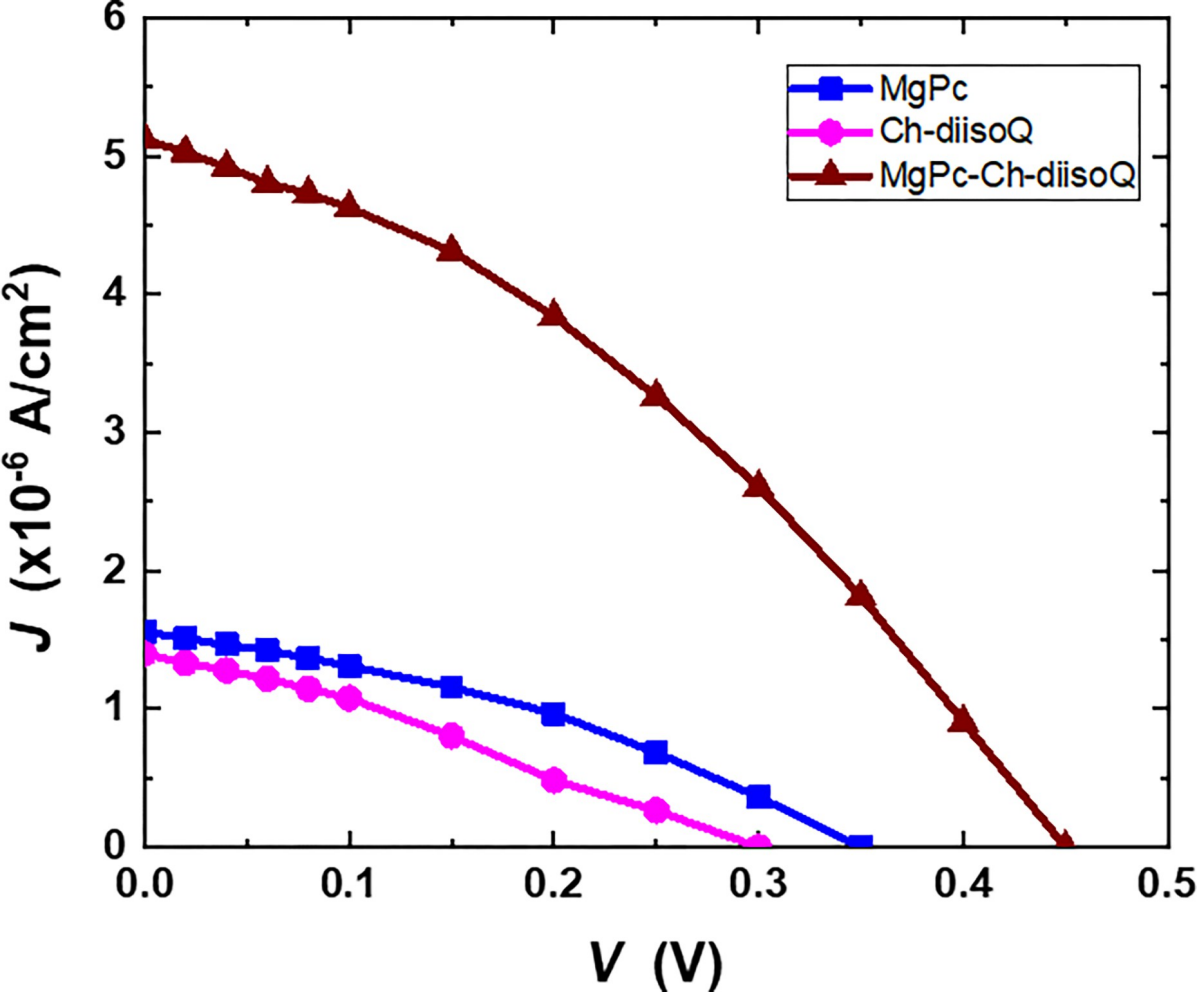
Load curves for FTO/MgPc/Al, FTO/Ch-diisoQ/Al, and FTO/MgPc-Ch-diisoQ/Al cells.

**Fig 12:**
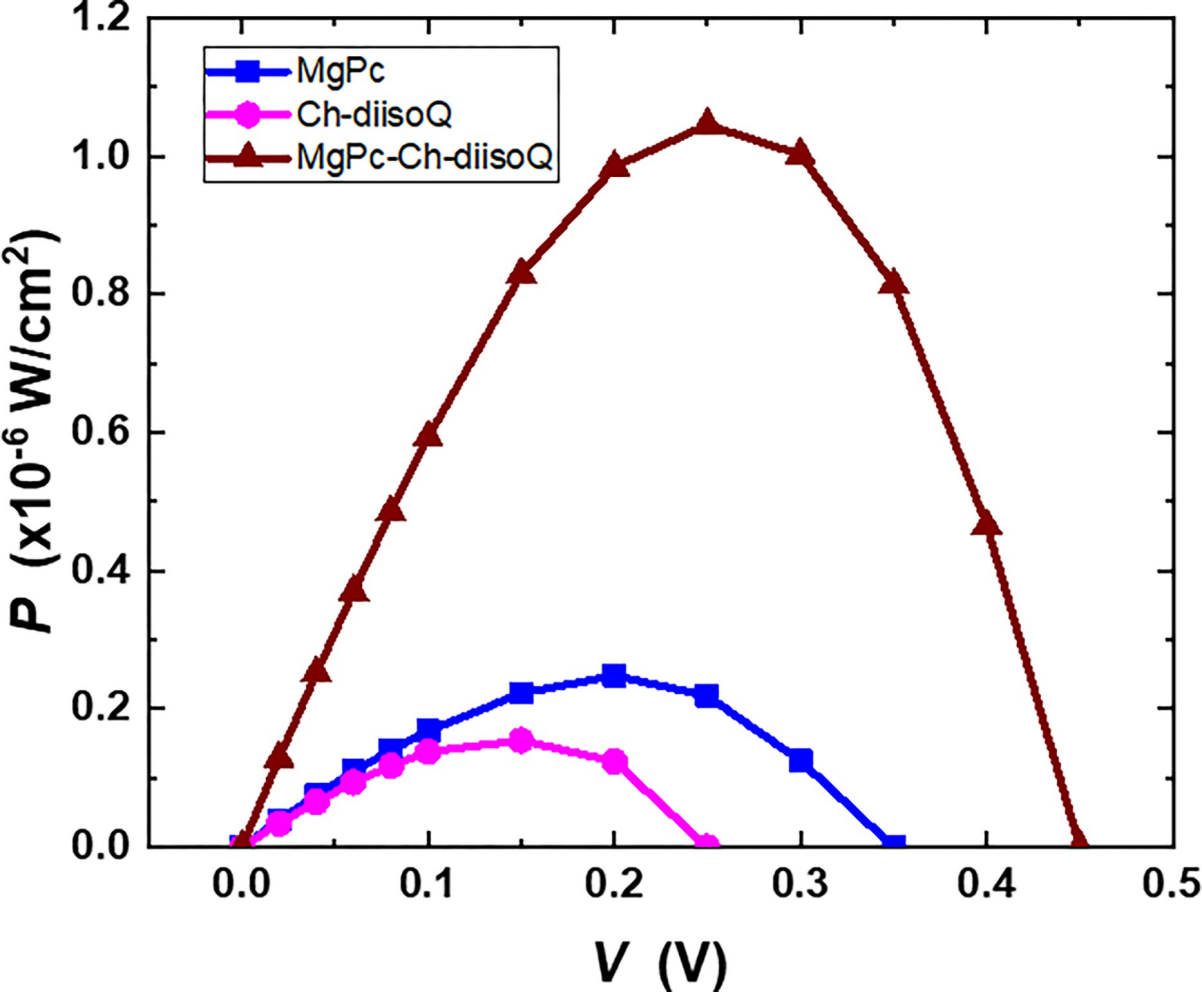
Power curves for FTO/MgPc/Al, FTO/Ch-diisoQ/Al, and FTO/MgPc-Ch-diisoQ/Al cells.

By connecting the cells to a load resistance that could be adjusted from 0 to infinity, the photocurrent density and power of MgPc/FTO, Ch-diisoQ/FTO, and MgPc-Ch-diisoQ/FTO cells were studied under illumination. Fig 11 shows the load curves for our investigated cells. This figure verifies that MgPc-Ch-diisoQ/FTO cells reveal the best response to the incident light. The Power curves for MgPc/FTO, Ch-diisoQ/FTO, and MgPc-Ch-diisoQ/FTO cells have been introduced in Fig 12. The extracted result of this figure shows the capability of using MgPc-Ch-diisoQ/FTO as candidate film for the design of solar cells. Photovoltaic parameters like short circuit photocurrent density (*J*_*sc*_) and open circuit voltage (*V*_*oc*_) can be calculated. Table 2 lists the parameters estimated from Fig 10 for the three cells. The fill factor (*FF*) values, which measure the approaching of the device to the ideal one, are given by relation [24]:

$$FF=\frac{J_{m}\;V_{m}}{J_{sc}\;V_{oc}}$$ (8)

where *J*_*m*_ and *V*_*m*_ are the current density and the voltage at the maximum power point. Table 2 shows the calculated *FF* values. The results show that the *FF* values are low, which can be attributed to either a high *R*_*s*_ of the organic layer or a low rate of separation of electron-hole pairs (excitons). The *I*_*sc*_ value of the MgPc-Ch-diisoQ/FTO cell is the greatest, which can be explained due to the increase of the electron mobility in the blend layer. Based on what is presented in Table 2, it is evident that an increased fill factor (*FF*) indicates a successful reduction in the energy losses experienced by the solar cell. This reduction consequently leads to a more efficient process of converting energy. Furthermore, a higher *J*_*sc*_ signifies that you have improved the absorption of sunlight and enhanced the generation of charge carriers within the cell, leading to greater current output. Finally, an increase in *V*_*oc*_ suggests that you have reduced the energy losses and improved the electrical characteristics of the cell, allowing it to generate a higher voltage under open-circuit conditions. By improving all three of these performance parameters in your solar cell research, you have successfully enhanced its overall efficiency. An efficient solar cell can capture a larger portion of the available sunlight, convert it into electricity more effectively, and ultimately provide a higher power output. This achievement represents a significant step forward toward advancing renewable energy technologies and making solar power more competitive and sustainable.

**Table 2:** Photovoltage parameters of MgPc, Ch-diisoQ, and MgPc/ Ch-diisoQ films.

	*V*_*oc*_ (V)	*J*_*sc*_ (×10^−6^ A/cm^2^)	FF
MgPc/FTO/Al	0.38	2.01	0.35
Ch-diisoQ /FTO/Al	0.25	1.79	0.33
MgPc /Ch-diisoQ /FTO/Al	0.45	6.57	0.49

## 4. Conclusions

In this study, we prepared three films of magnesium phthalocyanine (MgPc), chlorophenyl ethyl diisoquinoline (Ch-diisoQ), and a blend of MgPc-Ch-diisoQ. The three tested films are amorphous. The values of *E*_*g*_ of MgPc, Ch-diisoQ, and MgPc-Ch-diisoQ are 1.51, 1.08, and 1.58 eV, respectively. The blend film has three absorption bands at 800–600 nm, 600–400 nm, and 400–250 nm, with a wide range of light absorption, making it a promising material for solar cells. MgPc/FTO, Ch-diisoQ/FTO, and MgPc-Ch-diisoQ/FTO cells were fabricated by using a thermal coating unit of evaporation. The blend cell shows a higher rectification ratio, and the forward current density increases by 22%, suggesting the MgPc-Ch-diisoQ/FTO cell is an excellent rectification diode. The built-in voltage value of our investigated blend was about 0.92. The blend cell shows a short circuit photocurrent and the greatest open circuit voltage. The outcome of our research can nominate MgPc-Ch-diisoQ in organic solar cells.
